# Adaptive Noise Reduction Algorithm to Improve R Peak Detection in ECG Measured by Capacitive ECG Sensors

**DOI:** 10.3390/s18072086

**Published:** 2018-06-29

**Authors:** Minseok Seo, Minho Choi, Jun Seong Lee, Sang Woo Kim

**Affiliations:** 1 Department of Electrical Engineering, Pohang University of Science and Technology, Pohang 37673, Korea; seomseok@postech.edu (M.S.); junsunglee@postech.edu (J.S.L.); 2 Department of Creative IT Engineering and Future IT Innovation Laboratory, Pohang University of Science and Technology, Pohang 37673, Korea; minho17@postech.edu

**Keywords:** active noise cancellation, affine projection sign algorithm, noisy signal, motion artifacts, QRS interval, SNR

## Abstract

Electrocardiograms (ECGs) can be conveniently obtained using capacitive ECG sensors. However, motion noise in measured ECGs can degrade R peak detection. To reduce noise, properties of reference signal and ECG measured by the sensors are analyzed and a new method of active noise cancellation (ANC) is proposed in this study. In the proposed algorithm, the original ECG signal at QRS interval is regarded as impulsive noise because the adaptive filter updates its weight as if impulsive noise is added. As the proposed algorithm does not affect impulsive noise, the original signal is not reduced during ANC. Therefore, the proposed algorithm can conserve the power of the original signal within the QRS interval and reduce only the power of noise at other intervals. The proposed algorithm was verified through comparisons with recent research using data from both indoor and outdoor experiments. The proposed algorithm will benefit a noise reduction of noisy biomedical signal measured from sensors.

## 1. Introduction

The electrocardigram (ECG) is used to diagnose diseases, such as heart disease, by displaying bioinformation, such as R peak magnitude and interval [1,2]. Heart rate, which can be calculated using R peaks, often needs to be monitored for patients with diseases, as well as healthy individuals. To perform an ECG, Ag-AgCl electrodes must be attached to the patient’s body directly, which may be inconvenient in practice.

To overcome measurement limitations, capacitive ECG (cECG) sensors were developed to use capacitive coupling to measure voltage changes. Because ECGs in other intervals than QRS interval are relatively weak in power, the ECGs measured from the cECG sensors are mainly used to effectively detect R peaks, which can be used to derive a lot of medical information. Because of the convenience of cECG sensors, they are widely used in many situations where conventional ECG measurement is difficult but necessary [3,4,5]. For example, ECG measurement cannot be easily performed while driving, but evaluation of a driver may be necessary to prevent emergency situations that can be caused if a driver has a heart attack or falls asleep while driving. Therefore, cECG sensors could be mounted into the driver’s seat to obtain ECGs of drivers [6,7,8].

However, cECG sensors are sensitive to the noise caused by motion. As a result, accurate R peak detection is not possible. It is impossible to limit motion caused by breathing, vibrations, and various other actions of daily life; therefore, noise reduction via signal processing is necessary. Many methods for noise reduction have been suggested using bandpass filters [9], wavelet filters [10,11], independent component analysis [12], injection signals [13], spectro-temporal filtering [14], extended Kalman Filters [15], and active noise cancellation (ANC) [16,17,18]. Among them, adaptive filters, which are used in ANC, can effectively reduce noise that constantly changes by updating the filter weight adaptively as the noise changes. ANC has been studied to find a reference signals that can improve noise-reduction performance, but the commonly used reference signal that is obtained from acceleration sensors was not efficient in removing the noise from cECGs [17,18].

Recently, new reference signals obtained from additional cECG sensors were suggested to reflect the characteristics of the motion noise but not the bioinformation [6]. ANC using the new reference signal was verified to improve R peak detection by comparing the proposed algorithm to previous algorithms. Furthermore, reliability of the reference signal was shown by verifying the assumptions for ANC. However, the assumption for correlation with the reference noise was not clearly verified and it was sometimes wrong according to the subjects. Moreover, the adaptive filter of ANC was updated without considering that the power of the ECG within the QRS interval is much larger than the noise or the intermittence of the QRS interval. These problems caused a reduction in the power of the ECG and degraded R peak detection. Therefore, a new structure is needed for ANC to conserve the power of the ECG while reducing the power of the noise.

This study analyzes the properties of the ECG and the reference signal, and their effect on the update of the filter weight. As the filter weight within the QRS interval is updated as impulsive noise is added, we regard the measured ECG at the QRS interval to be impulsive noise. Accordingly, this paper proposes a new ANC structure using a robust variable step size affine projection sign algorithm (RVSS APA) that does not affect impulsive noise [19]. As a result, the power of the ECG signal within the QRS interval is conserved while the power of the noise is reduced to improve R peak detection.

## 2. Material

### 2.1. Measurement System

**Figure 1:**
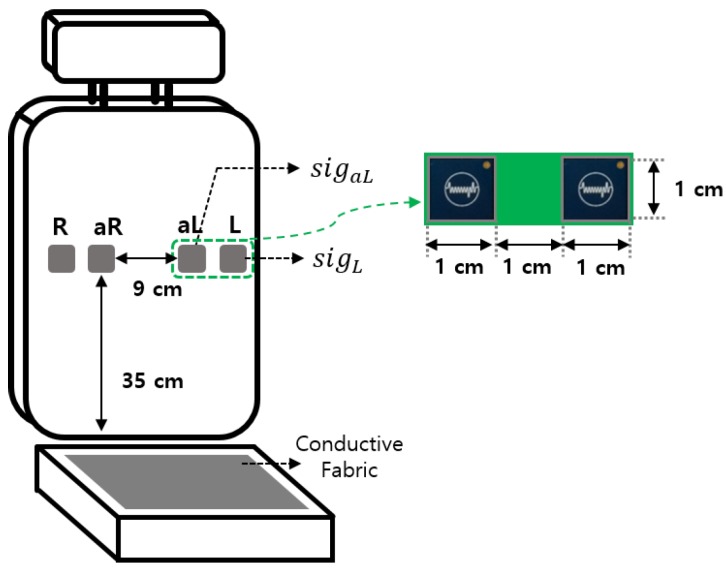
The measurement system.

In recent research [6], four cECG sensors were mounted in a row on the back of a chair and driver’s seat (Figure 1). Conductive fabric was placed on the bottom of the seat and connected to a driven right leg circuit to reduce the common noise component of the measured ECG. cECGs $sig_{L}$ and $sig_{R}$ were obtained from the two sensors outside in a row and additional cECGs $sig_{aL}$ and $sig_{aR}$ were obtained from two other sensors, which were close to the center. This study used signals that were defined and used in the existing research [6]. The difference between $sig_{L}$ and $sig_{R}$ is filtered through a band pass filter (BPF) with a 0.05–35 Hz pass-band; this was defined as a measured ECG ($ECG_{m}$):

(1) $$ECG_{m}=\mathrm{BPF}\left[sig_{L}-sig_{R}\right].$$

Two reference signals, $r_{L}$ and $r_{R}$, were obtained from the two sensors of each part as

(2) $$r_{L}=sig_{L}-sig_{aL},$$

(3) $$r_{R}=sig_{R}-sig_{aR}.$$

$r_{L}$ and $r_{R}$ were selected because they contain motion information, but do not have bioinformation [6]. Then, we used an NI 9205 (National Instruments, Austin, TX, USA) with 16 bits to convert the obtained analog signals to digital signals at 200 Hz.

### 2.2. Data Acquirement

The data were acquired from both indoor and outdoor experiments. Nineteen subjects participated in the indoor experiment.The participants were between 20 and 30 years old; one was female and the others were male. Ten subjects participated in the outdoor experiment. The participants were between 20 and 30 years old; all were male. All subjects wore cotton T-shirts during the experiments. During both experiments, cECGs were obtained from the cECG sensors. In addition, $ECG_{contact}$ was simultaneously obtained from conventional contact electrodes to obtain true R peaks.

During the indoor experiment, the subjects sat on the chair in which the measurement system had been installed. Then, they conducted the indoor experiment for 30 min according to following guidelines. For the first 5 min, the subjects remained stable without any movement to obtain an cECG without the noise. Leaning their backs against the cECG sensors, the subjects moved their bodies randomly to obtain an cECG with the noise for 25 min. The size and shape of the movement varied, and short breaks were given between movements. As a result, there was noise with a variety of power and frequency in the measured cECG, as can be assumed to be true for many situations.

During the outdoor experiment, the subjects sat in the driver’s seat, in which the measurement system had been installed (similar to the chair in the indoor experiment). The subjects were asked to drive along a course in the Pohang, Kyungbuk province of Korea. It took an average of 55 min for the participants to drive 31.8 km. The driving course consisted of 2.2 km of on-campus roads, 9.3 km of city roads, 9.2 km of national highway, and 11.1 km of highway. There were road signs and variations along the course, such as signal-induced stops, speed bumps, and unpaved roads. As a result, various types of noise were mixed into the measured cECG. In this experiment, the subjects were required to drive normally and not be concerned with leaning against the cECG sensors.

R peaks in the ECGs obtained during the experiments were detected using an R peak detection algorithm with the Pan and Tompkins algorithm [20] owing to ease of implementation and prior researches [6,16,21]. $ECG_{contact}$ was used to obtain true R peak positions and to evaluate the accuracy of obtained R peaks from the $ECG_{m}$.

## 3. Methods

### 3.1. Active Noise Cancellation

#### 3.1.1. ANC Using Affine Projection Algorithm

**Figure 2:**
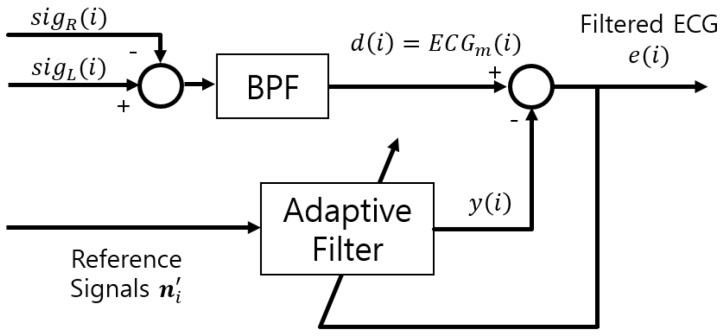
The block diagram of ANC.

ANC is used to remove noise from the measured signal [22,23,24] and its block diagram is depicted in (Figure 2). d(i) is a sequence of $ECG_{m}$ and equals s(i)+n(i), where *i* denotes the iteration. s(i) is the original ECG and n(i) is the noise from the motion of the subject and the vehicle. The reference signal $n_{i}^{\prime }$ is filtered through an adaptive filter to produce the output y(i). Then, the error signal e(i)=d(i)−y(i) is obtained and used as the output of ANC.

During ANC, noise can be cancelled when $n_{i}^{\prime }$ is correlated with n(i), and s(i) is not strongly correlated with n(i) and $n_{i}^{\prime }$ [6,22]. The expectation value of squared e(i) is obtained as

(4) $$\begin{array}{ccc} E\left[e^{2}\left(i\right)\right] & = & E\left[{\left(s\left(i\right)+n\left(i\right)-y\left(i\right)\right)}^{2}\right]\\ & = & E\left[s^{2}\left(i\right)\right]+E\left[{\left(n(i)-y(i)\right)}^{2}\right]+2E\left[s\left(i\right)\left(n(i)-y(i)\right)\right]\\ & = & E\left[s^{2}\left(i\right)\right]+E\left[{\left(n\left(i\right)-y\left(i\right)\right)}^{2}\right], \end{array}$$

where $E\left[\cdot \right]$ denotes the expectation. The weight of the adaptive filter is updated to minimize $E\left[e^{2}\left(i\right)\right]$ so that $\min\left\{E\left[e^{2}\left(i\right)\right]\right\}=E\left[s^{2}\left(i\right)\right]+\min\left\{E\left[n(i)-y(i)\right]\right\}$. As a result, n(i)−y(i) approaches zero and e(i) approximates s(i). The reduction of noise depends on the extent to which the correlation conditions are satisfied. Therefore, the selection of the reference signal is the most important step and has been researched to satisfy these conditions [6,17,18]. Reference noise $n_{i}^{\prime }$, determined using the differences between the cECGs from adjacent sensors, has been suggested to improve R peak detection by reducing the noise of $ECG_{m}$ [6]:

(5) $$n_{i}^{\prime }={\left[r_{L}\left(i\right),r_{L}\left(i-1\right),\ldots ,r_{L}\left(i-L+1\right),r_{R}\left(i\right),r_{R}\left(i-1\right),\ldots ,r_{R}\left(i-L+1\right)\right]}^{T},$$

where L denotes half the length of the filter weight and T denotes its transpose. In addition, an affine projection algorithm (APA) is used to update the weight of the adaptive filter. As $n_{i}^{\prime }$ itself is highly correlated and the correlation reduces the convergence rate of the filter weight [25], the APA can improve the convergence rate. The filter weight ${\hat{w}}_{i}={\left[{\hat{w}}_{1}\left(i\right),{\hat{w}}_{2}\left(i\right),\ldots ,{\hat{w}}_{2L}\left(i\right)\right]}^{T}$ of the APA is updated every iteration as follows:

(6) $$\begin{array}{ccc} U_{i} & = & \left[n_{i}^{\prime },n_{i-1}^{\prime },\ldots ,n_{i-P+1}^{\prime }\right],\\ e_{i} & = & d_{i}-U_{i}^{T}{\hat{w}}_{i-1},\\ d_{i} & = & {\left[d\left(i\right),d\left(i-1\right),\ldots ,d\left(i-P+1\right)\right]}^{T},\\ {\hat{w}}_{i} & = & {\hat{w}}_{i-1}+\mu U_{i}{\left(U_{i}^{T}U_{i}\right)}^{-1}e_{i}, \end{array}$$

where P is the projection order and μ is the step size of the APA. P determines the number of input vectors of $U_{i}$ and μ determines the convergence rate of the filter weight. The convergence rate is important in ANC because it determines the noise reduction performance when noise with different statistical characteristics is added. $e_{i}={\left[e_{1}\left(i\right),e_{2}\left(i\right),\ldots ,e_{P}\left(i\right)\right]}^{T}$ is the error vector and its first-tap error $e\left(i\right)=e_{1}\left(i\right)$ is the output sequence of ANC.

#### 3.1.2. Properties of Reference Signal and ECG

**Figure 3:**
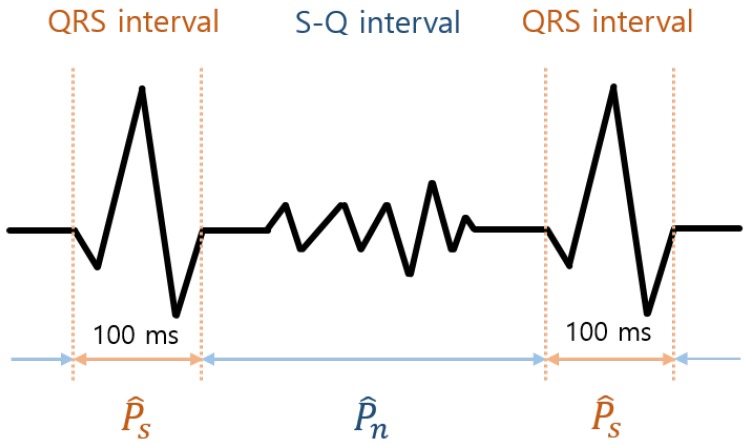
Definition of QRS interval, S-Q interval, ${\hat{P}}_{s}$, and ${\hat{P}}_{n}$ in the ECG.

**Figure 4:**
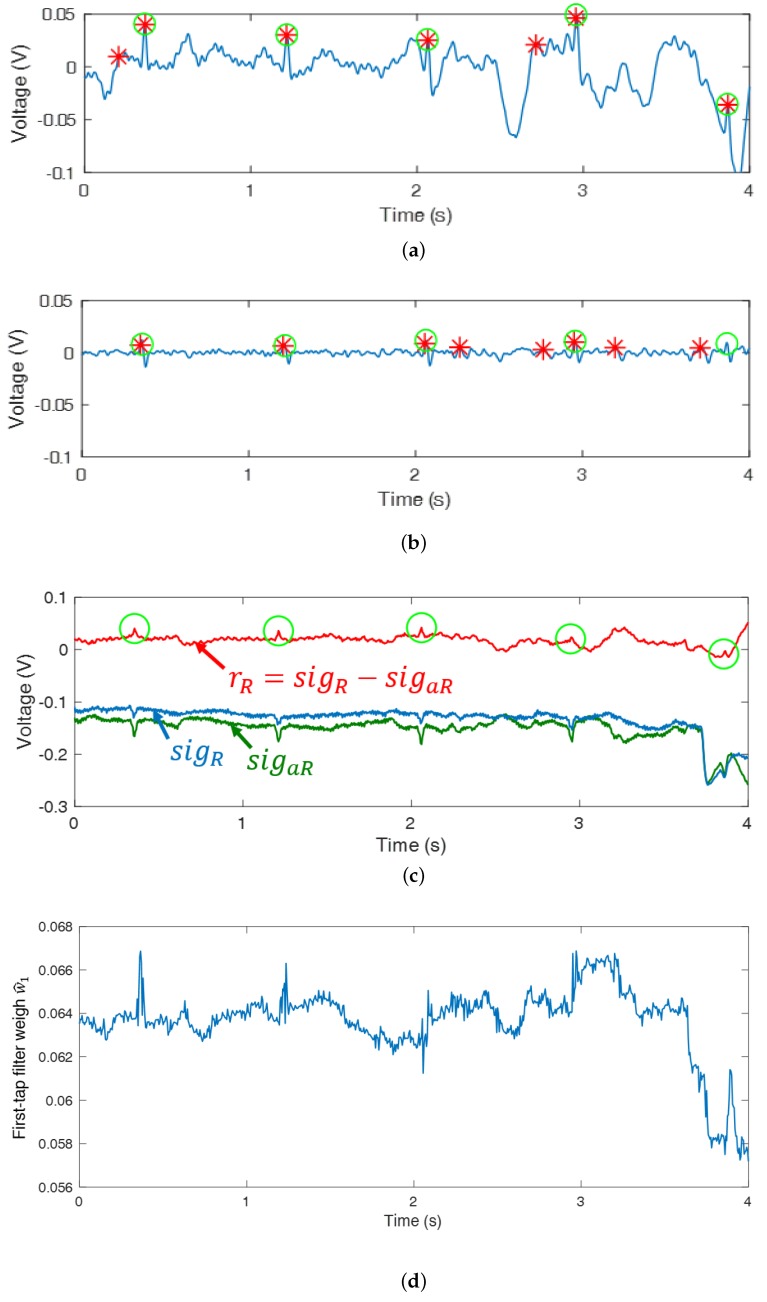
ECG filtered by the APA: (**a**) $ECG_{m}$, (**b**) ECG filter by the ANC with reduced power at the QRS interval, (**c**) $r_{R}$, $sig_{R}$, and $sig_{aR}$, and (**d**) the trajectory of the first-tap filter weight.

**Figure 5:**
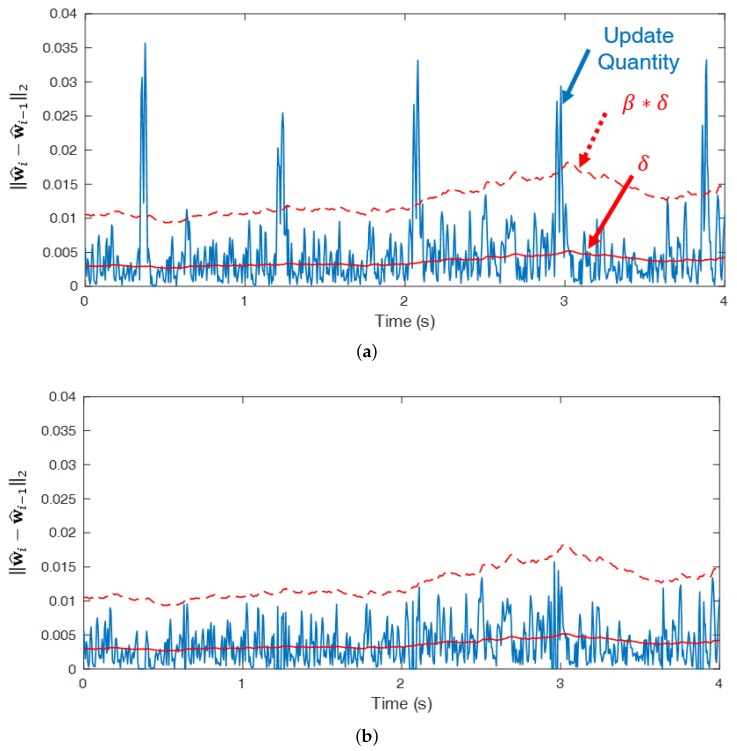
Update quantity of the filter weight: (**a**) update quantity by APA and (**b**) update quantity by the proposed algorithm using RVSS APA.

Although ANC reduces noise, it cannot improve R peak detection because it reduces the power of QRS interval (Figure 3) as well. To investigate this problem, s(i) has to be examined. In $r_{L}\left(i\right)$ and $r_{R}\left(i\right)$, s(i) is expected to be removed and only the signal correlated with the n(i) is expected to remain only motion information by subtracting each other’s cECG obtained from the adjacent cECG sensors. However, $r_{L}\left(i\right)$ and $r_{R}\left(i\right)$ are still correlated with s(i) because s(i) is not completely removed (Figure 4c). As a result, the correlation condition is not satisfied and the power of s(i) is reduced after filtering. In addition, the QRS interval of $ECG_{m}$ is intermittent and the power of s(i) at the QRS interval is large enough to ignore n(i). Therefore, within the interval, Equation (4) approximates $E\left[e^{2}\left(i\right)\right]\approx E\left[{\left(s\left(i\right)-y\left(i\right)\right)}^{2}\right]$. As a result, the filter weight $\hat{w}\left(i-1\right)$ is updated to remove s(i) from the output signal e(i) of the ANC even though the filter weight had been updated to reduce n(i) before the QRS interval. When $ECG_{m}$ (Figure 4a) is filtered through ANC, the power of the filtered ECG (Figure 4b) decreases at the QRS interval and more incorrect R peaks are detected, where the green o denotes the true R peak position and the red * denotes detected R peaks. Within the QRS interval, the first-tap filter weight dynamically changes (Figure 4d) and the updated quantity of the total filter weight increases (Figure 5a) compared to the S-Q interval (Figure 3). The filter weight has been continuously updated to remove the noise that exists in the S-Q interval, but s(i) within the QRS interval is large within the short time frame, causing the filter weight to be updated significantly to remove s(i).

To conserve the power of s(i) at the QRS interval, a dramatic update of the filter weight should be prevented by slowing the convergence rate. For a slow convergence rate, step size should be small; however, step size adjustment is hard because there is a serious trade-off problem. When the step size is small for slow convergence rate, the power of s(i) within QRS interval is conserved, but the power of n(i) is not reduced. In contrast, when the step size is large, the power of s(i) is reduced. In addition, the step size adjustment depends on the characteristics of each subject’s ECG.

To improve R peak detection with ANC, the filter weight at the QRS interval should be updated only for baseline noise reduction. Further, at the S-Q interval, which is defined as the entire interval except for the QRS interval (Figure 3), the filter weight should converge fast to reduce the noise. Based on the properties of s(i), we regard s(i) at the QRS interval as impulsive noise that requires a dramatic update of the filter weight when it is added. Therefore, robust algorithm against impulsive noise should be applied at the QRS interval, whereas the APA works for noise reduction at the other interval.

### 3.2. Robust Variable Step Size APA

Robust variable step size (RVSS) APA was introduced [19]. The RVSS APA updates its filter weight while switching the APA with the affine projection sign algorithm (APSA) [25]. The cost function of the APSA is $L_{1}$-norm of the aposteriori error vector $e_{p,i}=d\left(i\right)-U_{i}^{T}{\hat{w}}_{i}$ and the constraint is the update quantity of the filter weight $∥{\hat{w}}_{i}-{\hat{w}}_{i-1}{∥}_{2}^{2}\leq \delta^{2}$, where $\delta^{2}$ is a parameter for limited update quantity [26]. The update equation is obtained by solving the minimization problem:

(7) $${\hat{w}}_{i}={\hat{w}}_{i-1}+\frac{\delta U_{i}\cdot \mathrm{sgn}\left(e_{p,i}\right)}{\sqrt{\mathrm{sgn}\left(e_{p,i}\right)U_{i}^{T}U_{i}\mathrm{sgn}\left(e_{p,i}\right)}},$$

where sgn(·) denotes the sign function. APSA is robust against dynamic changes in filter weight, and its convergence rate is slow, so it should be used when update quantity by APA is large. Furthermore, the APA should be used to update the filter weight when the robustness of APSA is not necessary. In RVSS APA, ${\hat{w}}_{i-1}$ is updated by the APA when the update quantity of the APA $∥{\hat{w}}_{i}-{\hat{w}}_{i-1}{∥}_{2}={∥U_{i}{\left(U_{i}^{T}U_{i}\right)}^{-1}e_{i}∥}_{2}$ does not exceed threshold δ(i−1). Otherwise, ${\hat{w}}_{i-1}$ is updated by the APSA with δ(i−1) update quantity:

(8) $${\hat{w}}_{i}=\left\{\begin{array}{cc} {\hat{w}}_{i-1}+U_{i}{\left(U_{i}^{T}U_{i}\right)}^{-1}e_{i} & ,\mathrm{if}{∥U_{i}{\left(U_{i}^{T}U_{i}\right)}^{-1}e_{i}∥}_{2}<\delta \left(i-1\right)\\ {\hat{w}}_{i-1}+\frac{\delta \left(i-1\right)U_{i}\cdot \mathrm{sgn}\left(e_{i}\right)}{\sqrt{\mathrm{sgn}\left(e_{i}\right)U_{i}^{T}U_{i}\mathrm{sgn}\left(e_{i}\right)}} & ,\mathrm{otherwise}. \end{array}\right.$$

In the equation to update the APSA of the RVSS APA, $e_{p,i}$ is replaced with $e_{i}$. The δ(i) is recursively obtained as follows:

(9) $$\delta \left(i\right)=\alpha \delta \left(i-1\right)+\left(1-\alpha \right)\min\left\{\frac{\left|e(i)\right|}{∥n_{i}^{\prime }∥},\delta \left(i-1\right)\right\},$$

where α is a memory factor within [0,1] and generally close to one.

### 3.3. Proposed Algorithm with RVSS APA

At the QRS interval, the APSA is used to conserve power and reduce only the baseline noise because s(i) is considered impulsive noise. At the S-Q interval, APA is used to reduce the noise where n(i) is dominant. To satisfy with both goals, RVSS APA should be used with appropriate parameters, such as threshold and step sizes for the ECG-denoising system:

(10) $${\hat{w}}_{i}=\left\{\begin{array}{cc} {\hat{w}}_{i-1}+\mu_{1}U_{i}{\left(U_{i}^{T}U_{i}\right)}^{-1}e_{i} & ,\mathrm{if}{∥\mu_{1}U_{i}{\left(U_{i}^{T}U_{i}\right)}^{-1}e_{i}∥}_{2}<\beta \delta \left(i-1\right)\\ {\hat{w}}_{i-1}+\mu_{2}\left(i\right)\frac{U_{i}\cdot \mathrm{sgn}\left(e_{i}\right)}{\sqrt{\mathrm{sgn}\left(e_{i}\right)U_{i}^{T}U_{i}\mathrm{sgn}\left(e_{i}\right)}} & ,\mathrm{otherwise}, \end{array}\right.$$

where $\mu_{1}$ is the APA step size, $\mu_{2}\left(i\right)$ is the APSA step size, and βδ(i−1) is the threshold that determines when APSA is used.

#### 3.3.1. Step Size $\mu_{1}$ and $\mu_{2}$

The parameter $\mu_{1}$ decides the convergence rate of APA. The noise of the ECG is reduced by the APA especially within the S-Q interval. When $\mu_{1}$ becomes large and close to one, the noise is better reduced because the fast convergence rate can quickly reflect the constantly varying characteristic of the noise. However, when $\mu_{1}$ is too large, it can remove Q and S waves at the boundary of QRS interval. As a result, R peak detection can be degraded because Q and S waves are important to detect precise R peaks. Therefore, $\mu_{1}$ does not have to be too large.

In the APSA, $\mu_{2}$ equals δ(i−1) [19,25,26]. However, δ(i−1) is not small and constantly varies because the filter weight has been constantly updated due to the varying system. As a result, s(i) at the QRS interval can be removed with $\mu_{2}=\delta \left(i-1\right)$, which is not desirable. Therefore, $\mu_{2}$ has to be smaller than δ(i−1), but not zero. If $\mu_{2}$ is zero, the filter cannot adjust for the constantly varying system change. Therefore, $\mu_{2}$ is a function of δ(i) to reflect the system change:

(11) $$\mu_{2}=\gamma \delta \left(i-1\right).$$

where the γ is a scaling factor less than one.

#### 3.3.2. Threshold βδ

During every iteration, the update quantity of the APA is compared to βδ(i), and APSA is used instead of APA when the update quantity exceeds βδ(i). The update quantity of the filter weight is constantly changing due to experimental environment factors, such as ambient noise, the subjects, and experimental equipment. If δ(i) is constant, only the APSA is used so that the noise is not removed, or only the APA is used so that s(i) at the QRS interval is removed. The βδ(i) has to be at the boundary of QRS and S-Q intervals. Therefore, δ(i) has to be a function of the update quantity of the APA as βδ(i) is compared with only the update quantity of the APA as follows:

(12) $$\delta \left(i\right)=\left\{\begin{array}{cc} \alpha \delta \left(i-1\right)+\left(1-\alpha \right){∥\mu_{1}U_{i}{\left(U_{i}^{T}U_{i}\right)}^{-1}e_{i}∥}_{2} & ,\mathrm{if}\;{∥\mu_{1}U_{i}{\left(U_{i}^{T}U_{i}\right)}^{-1}e_{i}∥}_{2}<\beta \delta \left(i-1\right)\\ \delta (i-1) & ,\mathrm{otherwise}. \end{array}\right.$$

From this update, δ(i) can reflect the constantly varying update quantity of the APA. In addition, β is a scaling parameter to find only the boundary of the QRS and S-Q intervals. The update quantities of the APA at every iteration are shown in (Figure 5a) when $ECG_{m}$ is filtered through the proposed algorithm using RVSS APA. β should be within an appropriate range to locate the boundary and detect only the QRS interval. The update quantity is adjusted by the proposed algorithm (Figure 5b).

### 3.4. Postprocessing

In the research to developethe reference signal, a simple and reasonable postprocessing method is used [6] to prevent incorrect updates owing to exceptional situations that occur in practice. The filtered ECG was verified by two rules using two intervals from three consecutively detected R peaks and the power of ECG. When the filtered ECG is abnormal, its raw ECG is used instead. Owing to the simplicity and reliability of the postprocessing and the fair comparisons in following experiments, postprocessing is used in the proposed algorithm.

## 4. Experimental Results and Discussion

### 4.1. Performance Index

To verify the proposed algorithm with the new structure of the ANC, sensitivity (Se) and positive predictivity ($P^{+}$) were used as performance indices for detected R peaks. Se and $P^{+}$ is obtained by

(13) $$\mathrm{Se}=\frac{TP}{TP+FN}\times 100,$$

(14) $$P^{+}=\frac{TP}{TP+FP}\times 100.$$

A search window is defined as a 80-ms period centered on each true R peak [6]. True positive (TP) denotes the number of accurately detected R peaks within the search windows, false negative (FN) denotes the number of undetected R peaks within the search windows, and false positive (FP) denotes the number of detected R peaks outside the search windows. Se decreases when the peaks to be found are not detected and $P^{+}$ decreases when the wrong peaks are recognized as R peaks. In addition, their average is defined as $Avg=(\mathrm{Se}+P^{+})/2$.

Also, $\hat{\mathrm{SNR}}$ is used as a signal quality index. Because s(i) and n(i) are difficult to distinguish and obtain in practice, we assumed the original ECG power is the same as the power of the 100 ms centered around a true R peak as we considered the dominance of their power based on the quality index of ECG [27]. In the ECG measured by the cECG sensors, the complete PQRST wave is difficult to obtain, but complete QRS interval can be obtained. Therefore, the S-Q interval is considered the interval including only the noise. In this study, $\hat{\mathrm{SNR}}$ is defined as

(15) $$\hat{\mathrm{SNR}}=\frac{{\hat{P}}_{s}}{{\hat{P}}_{n}},$$

where ${\hat{P}}_{s}$ is the power of the 100-ms ECGs centered on the detected R peaks and ${\hat{P}}_{n}$ is the power of the ECG outside those 100-ms ECGs (Figure 3). $\hat{\mathrm{SNR}}$ decreases when the power at the QRS interval is not conserved by filtering.

### 4.2. Experimental Environment

To consider near-real-time noise reduction of the ECG, a 6-s processing window was set to process the length of the ECG, which the previously used 2-s data overlaps. The overlapped data enable R peaks at the boundary to be detected stably.

Se, $P^{+}$, and $\hat{\mathrm{SNR}}$, as obtained from $ECG_{m}$, $ECG_{APA}$, and $ECG_{RVSS}$ in the indoor and outdoor experiments, are shown in Table 1, where $ECG_{APA}$ is the ECG filtered by the ANC using APA [6] and $ECG_{RVSS}$ is the ECG filtered by the proposed algorithm using RVSS APA. The same postprocessing was also applied to ANC. For both experiments, the step size of ANC using APA was experimentally set to 0.05, which can lead to the best performance for the comparison. Furthermore, $\mu_{1}$, γ, and β were set as 0.4, 0.01, and 3.5. Se and $P^{+}$ were obtained using the entire length of each data set without exception. However, $\hat{\mathrm{SNR}}$ was obtained using only the initial 5-min length to ignore the baseline noise and noise during driving and motion because power is sensitive to large magnitudes of noise even if they do not last long. An increase in Avg compared to that of $ECG_{m}$ is defined as d_Avg and shown in each table to identify performance differences clearly.

The length of the data from the outdoor experiment is so long that the Avg increase seen is small. Therefore, the data were divided into 5 sections based on the roads that were driven. Se and $P^{+}$ are shown in Table 2. The 1st section is the initial 5-min section where every subject stayed stable, the 2nd section is the general road section in which many curves, speed bumps, and stops existed, the 3rd section is general road in which speed bumps and stops did not exist and fast driving was possible, the 4th section is a highway in which the noise constantly exited due to vehicle vibrations caused by faster speed, and the 5th section is national highway in which few curves and stops existed and fast driving was sometimes possible.

**Figure 6:**
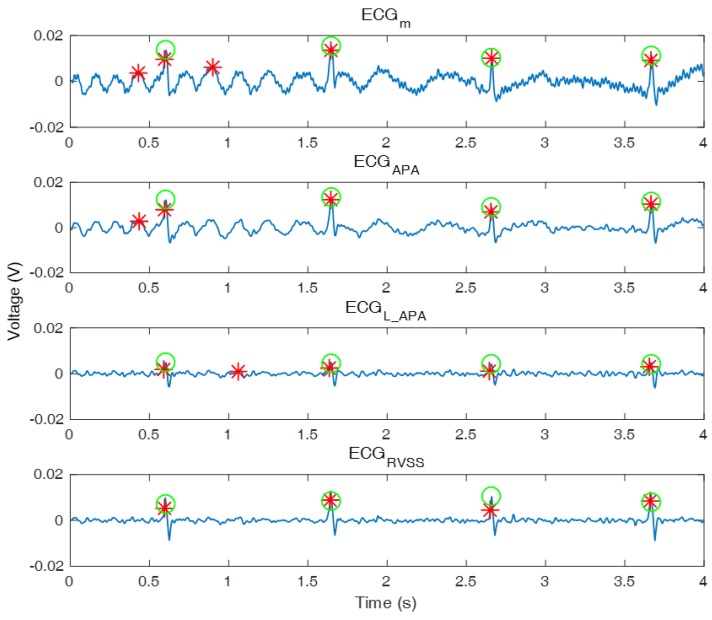
Comparison of $ECG_{RVSS}$ to $ECG_{m}$, $ECG_{APA}$ and $ECG_{\text{L\_APA}}$.

The $\hat{\mathrm{SNR}}$ of $ECG_{RVSS}$ was compared to that of $ECG_{m}$, $ECG_{APA}$ and $ECG_{\text{L\_APA}}$, which is the ECG filtered by the ANC using APA with a large step size of 0.4 in Table 3 to show the tradeoff in step size. Furthermore, $ECG_{m}$, $ECG_{APA}$, $ECG_{\text{L\_APA}}$, and $ECG_{RVSS}$ are plotted (Figure 6) to show the actual shape of the ECGs after being filtered.

### 4.3. Se and $P^{+}$ Comparison

In general, the Avg are used to evaluate the overall R peak detection performance, as both Se and $P^{+}$ are important. The Avg for $ECG_{RVSS}$ increased by 8.22 (mean) ± 5.55 (standard deviation)% in the indoor experiment and 6.28 ± 3.92% in the outdoor experiment, whereas the Avg for $ECG_{APA}$ increased by 5.58 ± 4.42% and 3.45 ± 2.32% in the indoor and outdoor experiment (Table 1). For indoor data 19, the Avg increased at most by 21.49%, and for outdoor data 4, the Avg increased at most by 14.11%. Actually, for several of the indoor and outdoor data sets, Se of $ECG_{RVSS}$ was less than that of $ECG_{APA}$, but $P^{+}$ was much larger, causing the Avg to be larger.

In addition, $P^{+}$ for $ECG_{RVSS}$ was 5.62 ± 5.00% larger than $P^{+}$ of $ECG_{APA}$ although Se for $ECG_{RVSS}$ was −0.33 ± 2.92% larger than Se of $ECG_{APA}$ in the indoor experiments. Likewise, $P^{+}$ for $ECG_{RVSS}$ was 5.48 ± 3.28% larger than that of $ECG_{APA}$ although Se for $ECG_{RVSS}$ was −0.20 ± 0.83% larger than that of $ECG_{APA}$ in outdoor experiments. The reason $P^{+}$ increased is that the proposed algorithm could filter ECG to reduce the power of the noise within the S-Q interval than the ANC using APA did. Thus, the proposed algorithm could prevent the detection of incorrect R peaks outside the search window, decreasing FP.

For more accurate results analysis (Table 2), the performance increase ratio (PIR) of each section were obtained and shown in the last column where

(16) $$\mathrm{PIR}=\frac{\text{d\_Avg}\;\mathrm{of}\;ECG_{RVSS}}{\text{d\_Avg}\;\mathrm{of}\;ECG_{APA}}.$$

**Figure 7:**
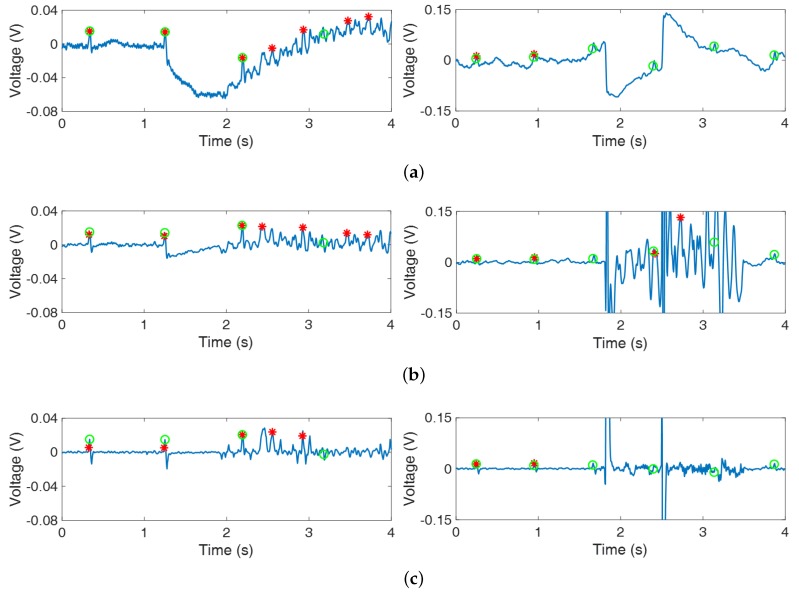
ECGs with abnormal noise filtered by the APA: (**a**) $ECG_{m}$ with abnormal noise, (**b**) $ECG_{APA}$, and (**c**) $ECG_{RVSS}$.

The two largest PIR were shown in Section 3 and Section 4. In those sections, the subjects could drive fast without curves, speed bumps, and stops so that only trivial and moderate noise occurred due to the vibration of the vehicle caused by fast driving and there was no abnormal noise. As a result, the proposed algorithm could reduce the noise and conserve the QRS interval compared to the ANC using APA. In the other sections, the PIR were lower than or similar to the PIR of the total section. There were curves, speed bumps, stops, and unpaved roads, so abnormal noise occurred. The severe vibration of the vehicle (i.e., driving on unpaved roads) resulted in noise which had statistical characteristics similar to QRS interval of original signal so that the QRS interval could not be distinguished (Figure 7a; left plot). Also, the separation of the body and sensor (i.e., steering, driving over speed bumps) resulted in large noise which caused wrong update of the filter weight (Figure 7; right plot). As a result, neither algorithms could reduce the abnormal noise (Figure 7b,c) and PIR did not increase. In conclusion, the performance depended on the type of noise. The proposed algorithm achieved improved results when moderate noise was mixed, but it showed the limitation that abnormal noise was not reduced.

### 4.4. ECGs and $\hat{SNR}$ Comparison

To investigate how much noise was removed and how much the QRS interval was preserved by filtering, ECGs and $\hat{\mathrm{SNR}}$ were compared. $ECG_{m}$, $ECG_{APA}$, $ECG_{\text{L\_APA}}$, and $ECG_{RVSS}$ from outdoor data 2 in were plotted in the same chart (Figure 6). Although all R peaks were detected in the $ECG_{m}$, two incorrect R peaks were detected due to motion noise. In $ECG_{APA}$, the motion noise was not removed sufficiently using the small step size even though the QRS-interval power was conserved. In contrast, in $ECG_{\text{L\_APA}}$, the motion noise was removed using a large step size, but the QRS-interval power was not conserved. Because of these problems, step size adjustment does not contribute significantly to improving SNR, which leads to performance degradation. As a result, the R peak detection algorithm detected wrong R peaks. However, the proposed algorithm removed the motion noise and conserved QRS-interval power. Then, SNR of $ECG_{RVSS}$ was larger than that of the other ECGs. As a result, only correct R peaks were detected without incorrect R peaks.

On average, $\hat{\mathrm{SNR}}$ of the $ECG_{RVSS}$ obtained from both experiments was larger than that of the $ECG_{m}$, $ECG_{APA}$, and $ECG_{\text{L\_APA}}$ (Table 3). For indoor data 8, 11, 13, and outdoor data 4, $\hat{\mathrm{SNR}}$ was smaller than $ECG_{APA}$. The quality of the $ECG_{m}$ to be filtered was abnormally noisy (Table 1), so the $ECG_{m}$ was hard to filter. In addition, $\hat{\mathrm{SNR}}$ of the $ECG_{APA}$ was larger than that of $ECG_{\text{L\_APA}}$ because $ECG_{\text{L\_APA}}$ reduced QRS-interval power more than noise power. This led to performance degradation so that the Avg for the $ECG_{\text{L\_APA}}$ were, on average, 79.35 ± 15.16% for the indoor experiment and 83.26 ± 5.96% for the outdoor experiment. When APA is used for noise reduction in ECG, its step size μ is normally selected between 0 and 1. Because there is a significant trade off with conservation of QRS interval and noise reduction, appropriate step size must be selected in the range according to experimental environments; in the 29 data of the both experiments, μ=0.05, on average, resulted in the largest Avg among μ=0.01,0.02,…,0.09,0.1,0.2, …, 1. However, in only two data, Avgs were the largest at μ=0.05, but Avgs were the largest at various size of μ in the other data. The difference between Avg at μ=0.05 and the largest Avg in each data was 0.59 ± 0.70%. However, the step size of the proposed algorithm has a significant role for only noise reduction, so the proposed algorithm with step size near 0.4 which is large enough to remove noise achieved high $\hat{\mathrm{SNR}}$ and improved R peak detection; in the 29 data, μ=0.4, on average, resulted in the largest Avg. Furthermore, in 11 data, Avgs were the largest at μ=0.4. Also, in most data, Avgs were the largest at μ near 0.4. The difference between Avg at μ=0.4 and the largest Avg in each data was 0.25 ± 0.29%. In conclusion, the step size of the ANC using APA must be selected differently depending on the experimental environments for the best performance. However, the best performance of the proposed algorithm is achieved when the step size is near 0.4 regardless of the experimental environments.

## 5. Conclusions

This study proposed a new ANC structure considering the properties of the ECG and the reference signal obtained from cECG sensors. The weight of the adaptive filter is significantly updated at the QRS interval because the correlation condition is not satisfied, and the original signal is regarded as impulsive noise within that interval. Because filter weight is updated if impulsive noise is present in the QRS interval, ANC using RVSS APA with appropriate parameters was suggested to be robust against the QRS-interval original signal. As a result, the raw ECG is filtered out as the power at the QRS interval is conserved and the power of the noise at the S-Q interval is reduced. $\hat{\mathrm{SNR}}$ of the filtered ECG increased, so improved R peak detection was achieved. Motion noise at S-Q interval was reduced; therefore, the inaccurate detection of R peaks in the raw ECG decreased. In the indoor and outdoor experiments, the proposed algorithm could reduce the noise of the raw ECG more than previous research of ANC using APA. As a result, Avg increased. In addition, the proposed algorithm achieves improved R peak detection when its step size is near 0.4 although there is a significant trade off with conservation of QRS interval and noise reduction in ANC using APA. It is because the proposed algorithm focuses on only noise reduction so step size which is large enough to reduce the noise can achieve improved R peak detection under various experimental environments.

However, the proposed algorithm could not reduce abnormal noise which causes wrong update of the filter weight. Then, the noise reduction performance depended on the types of noise. Furthermore, the proposed algorithm has a negative effect of losing other bioinformation (i.e., P wave, T wave) after adaptive filtering because the proposed algorithm focuses on the conservation of only QRS interval and noise reduction in S-Q interval containing P wave, and T wave for improved R peak detection. This negative effect can result in degraded signal quality. However, P wave and T wave are hardly measured by cECG sensors due to the limitation of the sensors and the measurement noise. Therefore, the proposed algorithm aimed at QRS interval and achieved improved R peak detection.

To improve R peak detection in our system, additional channel data can be used for noise reduction by mounting more sensors into the seat or by using the signal from the sensors already installed. As more channels can share the intervals among the R peaks, the QRS interval can be easily found and the power can be better conserved. More channel data can improve R peak detection by using a channel selection method with the proposed algorithm. The proposed algorithm can be applied to many applications measuring noisy biomedical signal.

## Figures and Tables

**Table 1:** Se, $P^{+}$, and Avg in the indoor and outdoor experiments.

	Data	${\mathrm{ECG}}_{m}$				${\mathrm{ECG}}_{\mathrm{APA}}$					${\mathrm{ECG}}_{\mathrm{RVSS}}$			
		Se	$P^{+}$	Avg		Se	$P^{+}$	Avg	d_Avg		Se	$P^{+}$	Avg	d_Avg
Indoor	1	80.57	70.00	75.28		87.42	82.50	84.96	9.68		86.26	84.97	85.61	10.33
	2	92.42	87.83	90.13		94.84	92.69	93.77	3.64		95.25	95.35	95.30	5.18
	3	95.83	95.98	95.91		96.38	97.11	96.74	0.84		96.34	97.38	96.86	0.95
	4	91.74	94.02	92.88		93.77	93.44	93.61	0.72		92.58	94.46	93.52	0.64
	5	91.76	82.69	87.23		94.64	89.91	92.28	5.05		94.79	94.79	94.79	7.56
	6	85.10	82.20	83.65		90.81	89.06	89.93	6.29		88.32	89.65	88.99	5.34
	7	91.48	86.93	89.20		95.16	90.53	92.85	3.64		94.20	94.20	94.20	5.00
	8	40.55	38.40	39.47		52.84	48.35	50.59	11.12		49.49	53.38	51.44	11.97
	9	92.11	69.62	80.87		93.55	78.61	86.08	5.21		93.73	88.38	91.05	10.19
	10	58.21	54.84	56.52		62.38	56.54	59.46	2.94		62.48	61.27	61.87	5.35
	11	52.13	39.96	46.05		54.00	40.42	47.21	1.16		63.93	61.86	62.89	16.84
	12	65.76	63.34	64.55		72.21	66.94	69.58	5.03		68.19	69.12	68.65	4.10
	13	63.77	49.24	56.50		69.19	52.69	60.94	4.44		64.82	58.12	61.47	4.97
	14	90.11	72.93	81.52		95.67	85.91	90.79	9.27		96.33	94.63	95.48	13.96
	15	83.87	70.05	76.96		84.48	71.03	77.75	0.79		84.48	82.59	83.54	6.58
	16	74.74	63.59	69.16		83.21	76.32	79.77	10.60		83.13	85.10	84.12	14.95
	17	73.37	84.40	78.88		82.61	87.61	85.11	6.23		82.18	91.18	86.68	7.80
	18	95.44	90.97	93.20		95.87	93.99	94.93	1.73		95.83	96.71	96.27	3.07
	19	81.30	62.92	72.11		92.70	86.77	89.73	17.62		93.13	94.07	93.60	21.49
	Mean	78.96	71.57	75.27		83.77	77.92	80.85	5.58		83.45	83.54	83.49	8.22
Outdoor	1	82.31	72.20	77.26		84.74	76.99	80.86	3.61		86.11	84.18	85.15	7.89
	2	90.17	74.99	82.58		90.09	78.20	84.14	1.56		90.86	84.56	87.71	5.13
	3	86.69	90.38	88.53		87.14	90.30	88.72	0.18		86.48	90.55	88.52	-0.02
	4	80.04	64.79	72.42		86.26	74.47	80.37	7.95		87.37	84.92	86.15	13.73
	5	85.24	70.70	77.97		88.35	77.79	83.07	5.10		88.30	87.43	87.87	9.89
	6	93.52	89.91	91.72		96.47	92.90	94.68	2.96		96.34	95.68	96.01	4.30
	7	81.13	79.93	80.53		82.94	83.15	83.05	2.52		82.35	84.74	83.55	3.02
	8	93.35	84.34	88.84		94.44	89.34	91.89	3.05		95.18	94.09	94.64	5.79
	9	88.32	79.14	83.73		91.99	87.38	89.69	5.96		92.49	92.76	92.63	8.90
	10	80.03	64.97	72.50		80.52	67.62	74.07	1.57		79.42	73.98	76.70	4.20
	Mean	86.08	77.14	81.61		88.29	81.81	85.05	3.45		88.49	87.29	87.89	6.28

**Table 2:** Avg, d_Avg, and PIR in each section from the outdoor experiment.

Section	${\mathrm{ECG}}_{m}$		${\mathrm{ECG}}_{\mathrm{APA}}$			${\mathrm{ECG}}_{\mathrm{RVSS}}$		PIR
	Avg		Avg	d_Avg		Avg	d_Avg	
1–5	81.61		85.05	3.45		87.89	6.28	1.82
1	87.12		89.83	2.71		91.79	4.67	1.72
2	82.08		85.99	3.91		88.32	6.23	1.59
3	82.62		84.33	1.71		86.52	3.90	2.28
4	78.11		81.40	3.29		84.88	6.77	2.06
5	81.50		84.12	2.62		86.35	4.85	1.85

**Table 3:** $\hat{\mathrm{SNR}}$ comparisons from the indoor and outdoor experiment.

	Data	$\hat{\mathrm{SNR}}$			
		${\mathrm{ECG}}_{m}$	${\mathrm{ECG}}_{\mathrm{APA}}$	${\mathrm{ECG}}_{\text{L\_APA}}$	${\mathrm{ECG}}_{\mathrm{RVSS}}$
Indoor	1	2.00	2.10	1.40	5.22
	2	2.87	3.41	2.21	7.96
	3	6.78	8.55	5.18	19.67
	4	5.91	6.95	4.27	25.82
	5	4.10	4.90	3.41	11.25
	6	2.58	3.93	3.19	10.14
	7	2.51	3.67	2.88	10.39
	8	1.47	0.90	0.44	0.69
	9	2.83	1.23	0.74	1.56
	10	7.41	9.40	6.51	26.95
	11	2.52	1.05	0.43	0.64
	12	1.21	1.08	0.64	1.13
	13	1.23	0.50	0.20	0.25
	14	2.02	2.35	1.38	3.72
	15	2.33	1.45	1.02	2.03
	16	1.85	1.49	0.88	2.09
	17	3.33	5.67	3.14	17.01
	18	2.86	3.57	2.52	10.90
	19	0.87	1.15	0.69	2.69
	Mean	2.98	3.33	2.16	8.43
Outdoor	1	5.15	2.88	2.81	12.58
	2	3.59	4.68	2.41	7.88
	3	6.49	11.13	4.25	29.91
	4	2.09	0.81	0.52	0.62
	5	2.56	2.79	1.96	5.86
	6	4.04	6.85	3.39	14.41
	7	2.54	3.66	2.29	6.43
	8	5.19	7.26	5.38	20.61
	9	3.64	5.69	3.41	14.50
	10	2.04	2.05	1.11	4.86
	Mean	3.73	4.78	2.75	11.77
